# Extranodal Anaplastic Large Cell Lymphoma Involving the Pancreas: A Rare Presentation

**DOI:** 10.7759/cureus.54037

**Published:** 2024-02-11

**Authors:** Haifa N Alsaleem, Ahmed S Almuzaini, Khaled Alnafisah, Nawaf Almutairi, Ammar M ALAmmari, Ahmed S AlOmar

**Affiliations:** 1 College of Medicine, Qassim University, Buraydah, SAU; 2 Gastroenterology, King Fahad Specialist Hospital, Buraydah, SAU; 3 Internal Medicine, King Fahad Specialist Hospital, Buraydah, SAU

**Keywords:** neoplasm of the pancreas, non hodgkin's lymphoma, extranodal lymphomas, pancreas lesion, t-cell lymphoma

## Abstract

Anaplastic large-cell lymphoma (ALCL) is a subtype of T-cell lymphoma. This disease mainly affects lymph nodes, although extranodal sites may also be involved. Lymphoma in the pancreas is a rare clinical entity whether it manifests as primary or extranodal involvement. We discuss an unusual case of a 29-year-old male patient who presented with epigastric pain and a right neck mass. The patient’s symptoms, physical examination, and laboratory tests prompted further investigation using imaging modalities such as CT, MRI, and endoscopic ultrasound, which revealed the presence of soft tissue masses in the right supraclavicular region and an ill-defined lesion within the pancreatic head. These findings eventually led to the identification of secondary extranodal pancreatic lymphoma. Fine needle biopsy (FNB) established an ultimate diagnosis of anaplastic lymphoma kinase (ALK)-positive ALCL.

## Introduction

Lymphoma is a neoplasm of the lymphoid tissues. It is classified into two types: Hodgkin lymphoma and non-Hodgkin lymphoma (NHL). NHL originates from B-cells or T-cells [1]. While NHL usually arises from the lymph nodes, it can also develop in extranodal sites, which include the stomach, skin, lungs, and, infrequently, the pancreas [2]. Peripheral T-cell lymphoma (PTCL) is a subtype of NHL and constitutes a rare and heterogeneous group of lymphoid malignancies that arise from mature or precursor T-cells. PTCLs account for approximately 15-20% of all non-Hodgkin lymphomas [3].

The World Health Organization (WHO) classifies PTCLs into many categories and subtypes. The classification mainly consists of three large groups: anaplastic large-cell lymphoma (ALCL), angioimmunoblastic T-cell lymphoma (AITL), and T-cell lymphoma-not otherwise specified (PTCL-NOS) [4]. ALCL is an aggressive lymphoma that frequently presents with advanced-stage disease (stage III-IV) and systemic symptoms, especially fever. It presents at an advanced stage in 65% of cases, and it manifests systemic symptoms in 75% of cases. It mostly affects lymph nodes, with extranodal involvement observed in 60% of cases. The most common extranodal sites of ALCL are the skin, subcutaneous tissue, bone, and lung [5].

ALCL is characterized by cells that are generally large with abundant cytoplasm and pleomorphic, often horseshoe-shaped, nuclei and wide CD30 expression. It is classified based on the presence or absence of a genetic abnormality involving the anaplastic lymphoma kinase (ALK) gene. ALK-positive ALCL is typically associated with a better prognosis than ALK-negative ALCL. ALC has three main subtypes: systemic ALCL, primary cutaneous ALCL, and breast implant-associated ALCL. Systemic ALCL can be either ALK-positive or ALK-negative, while primary cutaneous ALCL and breast implant-associated ALCL are typically ALK-negative [6]. Lymphoma in the pancreas is a very rare clinical entity; primary pancreatic lymphoma (PPL) represents <0.5% of pancreatic cancers and 1% of extranodal lymphomas [7].

## Case presentation

A 29-year-old male patient presented to the emergency department with a complaint of epigastric pain radiating to the back for three days. He had not passed stool for two days but was passing flatus. He reported no significant weight loss, fever, or night sweats. There was no history of gastrointestinal bleeding and vomiting. He had a history of a right neck mass three months prior, for which he was following up with oncology. His family history was unremarkable.

On physical examination, the patient had mild jaundice and a palpable mass in the right lower cervical region. There was no axillary or inguinal lymph node enlargement and no organomegaly. The remaining examination was unremarkable. Regarding laboratory tests, his complete blood count (CBC) was unremarkable. As for blood chemistry, he had elevated alanine transaminase (ALT), alkaline phosphatase (ALP), total bilirubin, gamma-glutamyl transferase (GGT), and lactate dehydrogenase (LDH), as demonstrated in Table 1. The observed malignancy, immunological, and infectious markers are shown in Table 2.

**Table 1:** Blood chemistry findings ALT: alanine transaminase; ALP: alkaline phosphatase; AST: aspartate aminotransferase; CK: creatine kinase; GGT: gamma-glutamyl transferase; LDH: lactate dehydrogenase; ESR: erythrocyte sedimentation rate

Test name	Results	Units
Albumin	41.1	g/L
ALT	250	U/L
ALP	199	U/L
AST	N/A	U/L
Amylase	54	U/L
Total bilirubin	71.0	umol/L
Calcium	2.24	mmol/L
Chloride	102	mmol/L
CK	77	U/L
GGT	262	U/L
LDH	355	U/L
Magnesium	0.81	mmol/L
Phosphorus	1.23	mmol/L
Potassium	4.20	mmol/L
Total protein	70.0	g/L
Random blood glucose	5.17	mmol/L
Sodium	140	mmol/L
Urea	2.9	mmol/L
Creatinine	78.4	umol/L
D-dimer	0.37	mg/dL
ESR	14	mm/1stHr

**Table 2:** Tumor markers and serological and immunological tests

Test name	Results	Units
Anti-LKM	Negative	%
Anti-Smith	Negative	%
Anti-mitochondrial antibody	Negative	%
Anti-PR3 (C-ANCA)	Negative	uIU/mL
Anti-MPO (P-ANCA)	Negative	%
Direct Coombs	Negative	%
Indirect Coombs	Negative	%
Thyroid-stimulating hormone (TSH)	0.497	uIU/mL
Alpha-fetoprotein (AFP)	1.30	IU/mL
Carcinoembryonic antigen (CEA)	2.24	ng/mL
CA 15-3	19-50	U/mL
CA 19-9	27.93	U/mL
CA 125	26.50	U/mL
Hepatitis B surface antigen (HBsAg)	Non-reactive	mL
HIV1, 2 Ab-Ag ELISA	Non-reactive	mL
Cytomegalovirus IgG	Negative	mL
Cytomegalovirus IgM	Negative	mL
Brucella abortus	Negative	%
Brucella melitensis	Negative	%
Hepatitis C antibody (HCVAb)	Non-reactive	%

**Figure 1:**
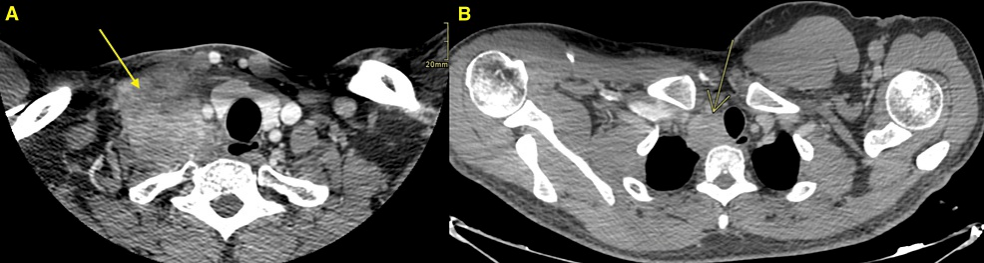
Chest CT The CT showed two right supraclavicular hypodense soft tissue masses; the larger one (A) measured about 5.4 x 5 x 5 cm at its anteroposterior (AP) x transverse (T) x craniocaudal (CC), respectively. The mass was seen abutting the thyroid gland and common carotid artery with the invasion of the proximal part of the internal jugular vein (IJV). The smaller mass (B) measured about 3.2 x 3.2 x 3.1 cm at its AP x T x CC, respectively. It encroached on the superior mediastinum pushing the trachea to the contralateral side and abutting the brachiocephalic artery CT: computed tomography

The patient underwent CT of the neck, chest, abdomen, and pelvis with contrast. Chest CT showed two right supraclavicular hypodense soft tissue masses (Figure 1).

**Figure 2:**
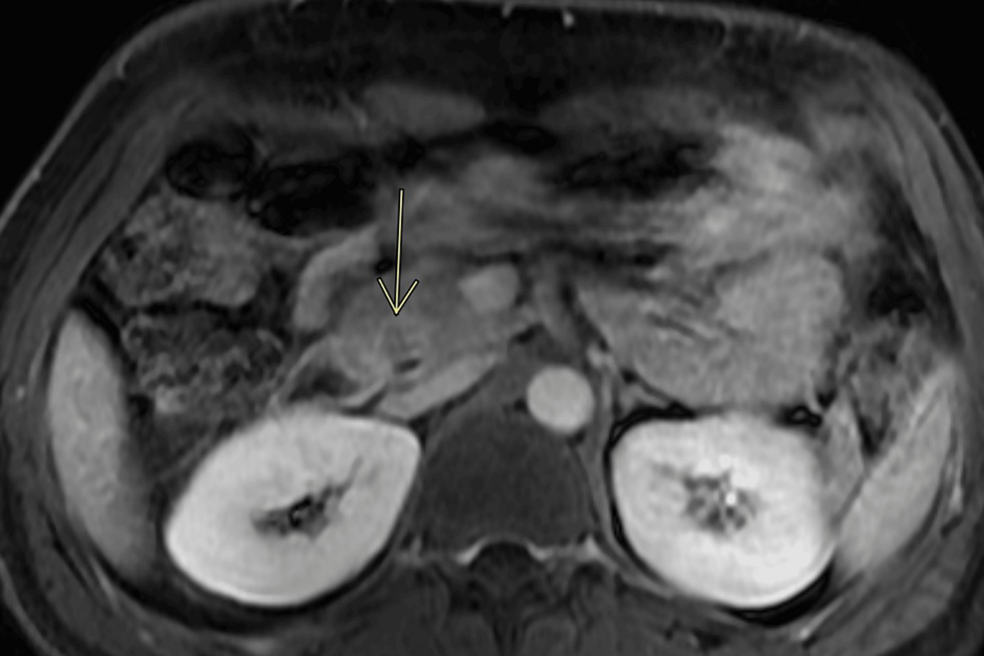
Pancreatic MRI The arrow shows a mass in the pancreatic head MRI: magnetic resonance imaging

The CT abdomen showed a dilated common bile duct (CBD) with a maximum diameter of 12.7 mm. The patient then underwent a pancreatic MRI to rule out periampullary lesions. It showed a 1.6 x 1.5 cm ill-defined lesion within the pancreatic head (Figure 2).

**Figure 3:**
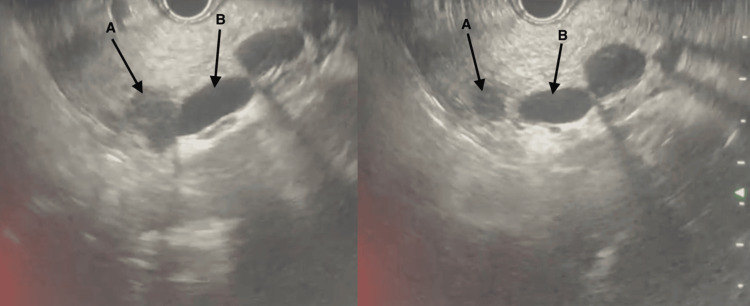
Endoscopic ultrasound The arrow (A) shows a hypoechoic mass at the head of the pancreas. The arrow (B) shows dilated CBD CBD: common bile duct

The patient also underwent endoscopic ultrasound (Figure 3), which showed a dilated CBD with a hypoechoic lesion at the head of the pancreas, and a fine-needle biopsy (FNB) was taken. The pancreatic head mass FNB showed T-cell lymphoma. Histology and immunohistochemistry were consistent with a diagnosis of ALCL, ALK-positive.

An endoscopic retrograde cholangiopancreatography (ERCP) was then done. It showed dilated CBD with a distal CBD stricture, but no filling defect or CBD stone was seen. A sphincterotomy was performed. Brush cytology was taken from the stricture and plastic stent was deployed in CBD with good biliary drainage. The bile duct brush/aspirate immunohistochemistry study revealed the following findings: the malignant cells were positive for LCA, ALK1, CD4, CD5, and CD30 (cytoplasmic and Golgi positive), but negative for CD20, PAX5, CKAE1/3, and synaptophysin.

After confirming the diagnosis, the patient received the first cycle of brentuximab CHP chemotherapy without any further complications, and he is currently in good health. He is scheduled to receive six more cycles in six months and will follow up with his remaining five cycles.

## Discussion

Lymphoma is a malignancy of the lymphatic system, which includes the lymph nodes, spleen, thymus, and bone marrow [2]. Lymphomas are broadly classified into two types: NHL, which accounts for 90% of cases, and Hodgkin, which makes up 10%. NHL is further divided into two groups according to the cell origin, either B-cells or T-cells. The B-cell presentation is much more common, and it approximately accounts for 90% of NHL [8]. The definitive cause of NHL is still unknown, but it has been hypothesized to be associated with various factors, such as infections (Epstein-Bart virus, hepatitis c virus, Helicobacter pylori infection, and human herpesvirus 8), environmental factors, immunodeficiency states, and chronic inflammation [1]. T-cell lymphomas represent about 10-15% of all NHL; they constitute a heterogeneous and often clinically aggressive group of lymphomas derived from mature T-cells [9].

ALCL is a subtype of T-cell lymphoma, and it accounts for approximately 2-3% of all NHL cases. While this disease mainly affects lymph nodes, extranodal sites may also be involved [10]. ALCL can be subdivided depending on the expression of ALK. The prevalence of ALK-positivity in patients with ALCL is 50-60%. ALK-positivity is usually seen in males younger than 35 years with more systemic symptoms, extranodal disease, and advanced-stage disease (III, IV), whereas ALK-negative patients tend to be older and are unlikely to present with extranodal disease. Age less than 60 years, normal serum LDH, a good performance status [Eastern Cooperative Oncology Group (ECOG) score <2], less than two extranodal sites of disease, and an International Prognostic Index score <4 are associated with better overall survival [11].

Confirming the ALK positivity is important because it denotes a significantly more favorable prognosis, in contrast to patients with ALK-negative ALCL. Also, the prognosis for ALK-positive and ALK-negative ALCL can be further classified based on CD56 positivity (neural cell-adhesion molecule), which demonstrates a significantly worse outcome when it is expressed in either ALCL subgroup [12]. The lymphatic system, being the source of lymphoma, typically initiates the disease in one of the many lymph nodes, leading to its spread throughout the immune system. This spread may either encompass various locations within the body or remain confined within the lymphatic system [13]. 

An early clinical indicator of NHL involves painless swelling of one or more of the 600+ lymph nodes in the body. However, crucial clinical attributes, known as "B symptoms," such as fever, night sweats, and unexplained weight loss, aid in distinguishing and diagnosing NHL from other cancers [13]. Extranodal NHL manifests in two forms. Primary extranodal disease arises when NHL originates in tissue within organs outside of the lymphatic system, like the pancreas [14]. On the other hand, extranodal lymphoma is termed secondary when there is lymph node involvement or when there are multiple extranodal sites. While secondary pancreatic involvement is more common than PPL, overall pancreatic involvement is rare [15].

Our case was categorized as secondary extranodal pancreatic lymphoma based on the presence of lymph node metastasis and the involvement of surrounding organs. PPL could be distinguished from secondary by applying the Dawson/Behrns criteria. The PPL must fulfill the following criteria: the absence of superficial or mediastinal adenopathy, normal leukocyte count, and confined peripancreatic disease without the involvement of the liver and spleen [16,17]. Another way to differentiate between them is based on the location of the lesion, as primary pancreatic malignancies typically arise from the head of the pancreas [16].

Pancreatic lymphoma symptoms and its initial imaging findings closely resemble those of other pancreatic tumors, which makes it difficult to diagnose. Several features differentiate extranodal lymphoma from adenocarcinoma of the pancreas, such as the size of the mass and its location. Laboratory values and biopsies are very important in the diagnosis and for initiating appropriate management, especially since treatment for lymphoma is highly different from that for primary pancreatic lesions [16].

## Conclusions

The case report highlights the diverse and often nonspecific clinical manifestations of lymphomas and the importance of considering lymphoma in the differential diagnosis of patients presenting with atypical symptoms and laboratory findings. Furthermore, the identification of specific subtypes of lymphoma, such as ALCL, and the characterization of their molecular and immunohistochemical features, such as ALK positivity, are crucial for determining the prognosis and guiding treatment decisions. The report also underscores the rarity of secondary extranodal pancreatic lymphoma and emphasizes the need for a comprehensive diagnostic approach, the challenges faced in their diagnosis, and the importance of a multidisciplinary approach involving clinical, laboratory, and imaging assessments to accurately identify and characterize these malignancies. It also sheds light on the significance of understanding the unique features of lymphoma subtypes to optimize patient management and improve outcomes.
